# Training Feedforward Neural Networks Using Symbiotic Organisms Search Algorithm

**DOI:** 10.1155/2016/9063065

**Published:** 2016-12-25

**Authors:** Haizhou Wu, Yongquan Zhou, Qifang Luo, Mohamed Abdel Basset

**Affiliations:** ^1^College of Information Science and Engineering, Guangxi University for Nationalities, Nanning 530006, China; ^2^Key Laboratory of Guangxi High Schools Complex System and Computational Intelligence, Nanning 530006, China; ^3^Faculty of Computers and Informatics, Zagazig University, Zagazig, Egypt

## Abstract

Symbiotic organisms search (SOS) is a new robust and powerful metaheuristic algorithm, which stimulates the symbiotic interaction strategies adopted by organisms to survive and propagate in the ecosystem. In the supervised learning area, it is a challenging task to present a satisfactory and efficient training algorithm for feedforward neural networks (FNNs). In this paper, SOS is employed as a new method for training FNNs. To investigate the performance of the aforementioned method, eight different datasets selected from the UCI machine learning repository are employed for experiment and the results are compared among seven metaheuristic algorithms. The results show that SOS performs better than other algorithms for training FNNs in terms of converging speed. It is also proven that an FNN trained by the method of SOS has better accuracy than most algorithms compared.

## 1. Introduction

Artificial neural networks (ANNs) [1] are mathematical models and have been widely utilized for modeling complex nonlinear processes. As one of the powerful tools, ANNs have been employed in various fields, like time series prediction [2], classification [3, 4], pattern recognition [5–7], system identification and control [8], function approximation [9], signal processing [10], and so on [11, 12].

There are different types of ANNs proposed in the literature: feedforward neural networks (FNNs) [13], Kohonen self-organizing network [14], radial basis function (RBF) [15–17], recurrent neural network [18], and spiking neural networks [19]. In fact, feedforward neural networks are the most popular neural networks in practical applications. Training process is one of the most important aspects for neural networks. In this process, the goal is to achieve the minimum cost function defined as a mean squared error (MSE) or a sum of squared error (SSE) by the means of finding the best combination of connection weights and biases. In general, training algorithms can be classified into two groups: gradient-based algorithms versus stochastic search algorithms. The most widely applied gradient-based training algorithms are backpropagation (BP) algorithm [20] and its variants [21]. However, in complex nonlinear problems, these two algorithms suffer from some shortcomings, such as highly depending on the initial solution, which subsequently impact on the convergence of the algorithm and easily get trapped into local optima. On the other hand, stochastic search methods like metaheuristic algorithms were proposed by researchers as alternatives to gradient-based methods for training FNNs. Metaheuristic algorithms are proved to be more efficient in escaping from local minima for optimization problems.

Various metaheuristic optimization methods have been used to train FNNs. GA, inspired by Darwinians' theory of evolution and natural selection [22], is one of the earliest methods for training FNNs that proposed by Montana and Davis [23]. The results indicate that GA is able to outperform BP when solving real and challenging problems. Shaw and Kinsner presented a method called chaotic simulated annealing [24, 25], which is superior in escaping from local optima for training multilayer FNNs. Zhang et al. proposed a hybrid particle swarm optimization-backpropagation algorithm for feedforward neural network training. In their research, a heuristic way was adopted to give a transition from particle swarm search to gradient descending search [26]. In 2012, Mirjalili et al. proposed a hybrid particle swarm optimization (PSO) and gravitational search algorithm (GSA) [27] to train FNNs [28]. The results showed that PSOGSA outperforms both PSO and GSA in terms of converging speed and avoiding local optima and has better accuracy than GSA in the training process. In 2014, a new metaheuristic algorithm called centripetal accelerated particle swarm optimization (CAPSO) was employed by Beheshti et al. to evolve the accuracy in training ANN [29]. Recently, several other metaheuristic algorithms are applied on the research of NNs. In 2014, Pereira et al. introduced social-spider optimization (SSO) to improve the training phase of ANN with multilayer perceptrons and validated the proposed approach in the context of Parkinson's disease recognition [30]. Uzlu et al. applied the ANN model with the teaching-learning-based optimization (TLBO) algorithm to estimate energy consumption in Turkey [31]. In 2016, Kowalski and Łukasik invited the krill herd algorithm (KHA) for learning an artificial neural network (ANN), which has been verified for the classification task [32]. In 2016, Faris et al. employed the recently proposed nature-inspired algorithm called multiverse optimizer (MVO) for training the feedforward neural network. The comparative study demonstrates that MVO is very competitive and outperforms other training algorithms in the majority of datasets [33]. Nayak et al. proposed a firefly based higher order neural network for data classification for maintaining fast learning and avoids the exponential increase of processing units [34]. Many other metaheuristic algorithms, like ant colony optimization (ACO) [35, 36], Cuckoo Search (CS) [37], Artificial Bee Colony (ABC) [38, 39], Charged System Search (CSS) [40], Grey Wolf Optimizer (GWO) [41], Invasive Weed Optimization (IWO) [42], and Biogeography-Based Optimizer (BBO) [43] have been adopted for the research of neural network.

In this paper, a new method of symbiotic organisms search (SOS) is used for training FNNs. Symbiotic organisms search [44], proposed by Cheng and Prayogo in 2014, is a new swarm intelligence algorithm simulating the symbiotic interaction strategies adopted by organisms to survive and propagate in the ecosystem. And the algorithm has been applied to resolve some engineering design problems by scholars. In 2016, Cheng et al. researched on optimizing multiple-resources leveling in multiple projects using discrete symbiotic organisms search [45]. Eki et al. applied SOS to solve the capacitated vehicle routing problem [46]. Prasad and Mukherjee have used SOS for optimal power flow of power system with FACTS devices [47]. Abdullahi et al. proposed SOS-based task scheduling in cloud computing environment [48]. Verma et al. investigated SOS for congestion management in deregulated environment [49]. Time-cost-labor utilization tradeoff problem was solved by Tran et al. using this algorithm [50]. Recently, in 2016, more and more scholars get interested in the research of the SOS algorithm. Yu et al. applied two solution representations to transform SOS into an applicable solution approach for the capacitated vehicle and then apply a local search strategy to improve the solution quality of SOS [51]. Panda and Pani presented hybrid SOS algorithm with adaptive penalty function to solve multiobjective constrained optimization problems [52]. Banerjee and Chattopadhyay presented a novel modified SOS to design an improved three-dimensional turbo code [53]. Das et al. used SOS to determine the optimal size and location of distributed generation (DG) in radial distribution network (RDN) for the reduction of network loss [54]. Dosoglu et al. utilized SOS for economic/emission dispatch problem in power systems [55].

The structure of this paper is organized as follows.  Section 2 gives a brief description of feedforward neural network; Section 3 elaborates the symbiotic organisms Search and Section 4 describes the SOS-based trainer and how it can be used for training FNNs in detail. In Section 5, series of comparison experiments are conducted; our conclusion will be given in Section 6.

## 2. Feedforward Neural Network

**Figure 1:**
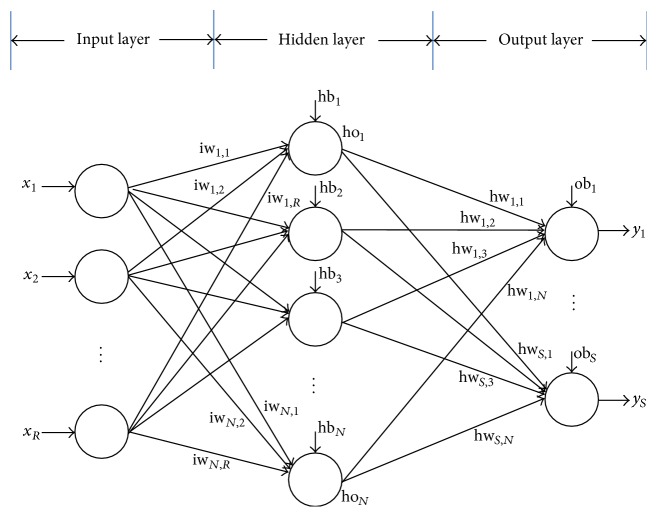
A feedforward network with one hidden layer.

In the artificial neural network, the feedforward neural network (FNN) was the simplest type which consists of a set of processing elements called “neurons” [33]. In this network, the information moves in only one direction, forward, from the input layer, through the hidden layer and to the output layer. There are no cycles or loops in the network. An example of a simple FNN with a single hidden layer is shown in Figure 1. As shown, each neuron computes the sum of the inputs weight at the presence of a bias and passes this sum through an activation function (like sigmoid function) so that the output is obtained. This process can be expressed as (1) and (2).

(1) $$\begin{array}{c} h_{j}=\overset{R}{\underset{i=1}{\sum }}{iw}_{j,i}x_{i}+{hb}_{j}, \end{array}$$

where iw_*j*,*i*_ is the weight connected between neurons *i* = (1,2,…, *R*) and *j* = (1,2,…, *N*), hb_*j*_ is a bias in hidden layer, *R* is the total number of neurons in input layer, and *x*_*i*_ is the corresponding input data.

Here, the S-shaped curved sigmoid function is used as the activation function, which is shown in

(2) $$\begin{array}{c} f_{\left(x\right)}=\frac{1}{1+e^{-x}}. \end{array}$$

Therefore, the output of the neuron in hidden layer can be described as in

(3) $$\begin{array}{c} {ho}_{j}=f_{j}\left(\;h_{j}\;\right)=\frac{1}{\left(1+e^{-h_{j}}\right)}. \end{array}$$

In the output layer, the output of the neuron is shown in

(4) $$\begin{array}{c} y_{k}=f_{k}\left(\;\overset{N}{\underset{j=1}{\sum }}{hw}_{k,j}{ho}_{j}+{ob}_{k}\;\right), \end{array}$$

where hw_*j*,*i*_ is the weight connected between neurons *j* = (1,2,…, *N*) and *k* = (1,2,…, *S*), ob_*k*_ is a bias in output layer, *N* is the total number of neurons in hidden layer, and *S* is the total number of neurons in output layer.

The training process is carried out to adjust the weights and bias until some error criterion is met. Above all, one problem is to select a proper training algorithm. Also, it is very complex to design the neural network because many elements affect the performance of training, such as the number of neurons in hidden layer, interconnection between neurons and layer, error function, and activation function.

## 3. Symbiotic Organisms Search Algorithm

Symbiotic organisms search [44] stimulates symbiotic interaction relationship that organisms use to survive in the ecosystem. Three phases, mutualism phase, commensalism phase, and parasitism phase, stimulate the real-world biological interaction between two organisms in ecosystem.

### 3.1. Mutualism Phase

Organisms engage in a mutuality relationship with the goal of increasing mutual survival advantage in the ecosystem. New candidate organisms for *X*_*i*_ and *X*_*j*_ are calculated based on the mutuality symbiosis between organism *X*_*i*_ and *X*_*j*_, which is modeled in (5) and (6).

(5) Xinew=Xi+αXbest−Mutual_Vector∗BF1

(6) Xjnew=Xj+βXbest−Mutual_Vector∗BF2

(7) Mutual_Vector=Xi+Xj2,

where BF_1_ and BF_2_ are benefit factors that are determined randomly as either 1 or 2. These factors represent partially or fully level of benefit to each organism. *X*_best_ represents the highest degree of adaptation organism. *α* and *β* are random number in [0,1]. In (7), a vector called “Mutual_Vector” represents the relationship characteristic between organisms *X*_*i*_ and *X*_*j*_.

### 3.2. Commensalism Phase

One organism obtains benefit and does not impact the other in commensalism phase. Organism *X*_*j*_ represents the one that neither benefits nor suffers from the relationship and the new candidate organism of *X*_*i*_ is calculated according to the commensalism symbiosis between organisms *X*_*i*_ and *X*_*j*_ which is modeled in

(8) $$\begin{array}{c} X_{inew}=X_{i}+\delta \left(\;X_{best}-X_{j}\;\right), \end{array}$$

where *δ* represents a random number in [−1, 1]. And *X*_best_ is the highest degree of adaptation organism.

### 3.3. Parasitism Phase

One organism gains benefit but actively harms the other in the parasitism phase. An artificial parasite called “Parasite_Vector” is created in the search space by duplicating organism *X*_*i*_ and then modifying the randomly selected dimensions using a random number. Parasite_Vector tries to replace another organism *X*_*j*_ in the ecosystem. According to Darwin's evolution theory, “only the fittest organisms will prevail”; if Parasite_Vector is better, it will kill organism *X*_*j*_ and assume its position; else *X*_*j*_ will have immunity from the parasite and the Parasite_Vector will no longer be able to live in that ecosystem.

## 4. SOS for Train FNNs

In this paper, symbiotic organisms search is used as a new method to train FNNs. The set of weights and bias is simultaneously determined by SOS in order to minimize the overall error of one FNN and its corresponding accuracy by training the network. This means that the structure of the FNN is fixed.  Figure 3 shows the flowchart of training method SOS, which is started by collecting, normalizing, and reading a dataset. Once a network has been structured for a particular application, including setting the desired number of neurons in each layer, it is ready for training.

### 4.1. The Feedforward Neural Networks Architecture

When implementing a neural network, it is necessary to determine the structure based on the number of layers and the number of neurons in the layers. The larger the number of hidden layers and nodes, the more complex the network will be. In this work, the number of input and output neurons in MLP network is problem-dependent and the number of hidden nodes is computed on the basis of Kolmogorov theorem [56]: Hidden = 2 × Input + 1. When using SOS to optimize the weights and bias in network, the dimension of each organism is considered as *D*, shown in

(9) D=Input×Hidden+Hidden×Output+Hiddenbias+Outputbias,

where Input, Hidden, and Output refer to the number of input, hidden, and output neurons of FNN, respectively. Also, Hidden_bias_ and Output_bias_ are the number of biases in hidden and output layers.

### 4.2. Fitness Function

In SOS, every organism is evaluated according to its status (fitness). This evaluation is done by passing the vector of weights and biases to FNNs; then the mean squared error (MSE) criterion is calculated based on the prediction of the neural network using the training dataset. Through continuous iterations, the optimal solution is finally achieved, which is regarded as the weights and biases of a neural network. The MSE criterion is given in (10) where *y* and $\hat{y}$ are the actual and the estimated values based on proposed model and *R* is the number of samples in the training dataset.

(10) $$\begin{array}{c} MSE=\frac{1}{R}\overset{R}{\underset{i=1}{\sum }}{\left(\;y-\hat{y}\;\right)}^{2}. \end{array}$$

### 4.3. Encoding Strategy

**Figure 2:**
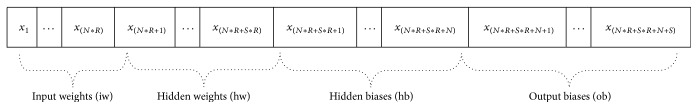
The vector of training parameters.

**Figure 3:**
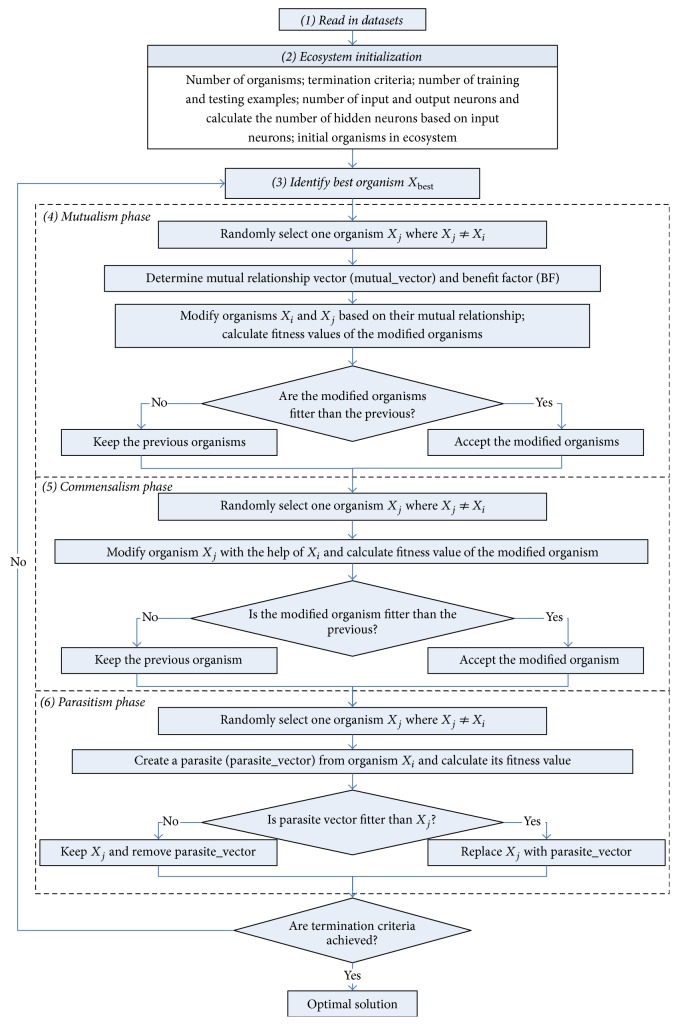
Flowchart of SOS algorithm.

According to [57], the weights and biases of FNNs for every agent in evolutionary algorithms can be encoded and represented in the form of vector, matrix, or binary. In this work, the vector encoding method is utilized. An example of this encoding strategy for FNN is provided as shown in Figure 2.

During the initialization process, *X* = (*X*_1_, *X*_2_,…, *X*_*N*_) is set on behalf of the* N* organisms. Each organism *X*_*i*_ = {iw, hw, hb, ob}  (*i* = 1,2,…, *N*) represents complete set of FNN weights and biases, which is converted into a single vector of real number.

### 4.4. Criteria for Evaluating Performance

Classification is used to understand the existing data and to predict how unseen data will behave. In other words, the objective of data classification is to classify the unseen data in different classes on the basis of studying the existing data. For the classification problem, in addition to MSE criterion, accuracy rate was used. This rate measures the ability of the classifier by producing accurate results which can be computed as follows:

(11) $$\begin{array}{c} \text{Accuracy}=\frac{\tilde{N}}{N}, \end{array}$$

where $\tilde{N}$ represents the number of correctly classified objects by the classifier and *N* is the number of objects in the dataset.

## 5. Simulation Experiments

This section presents a comprehensive analysis to investigate the efficiency of the SOS algorithm for training FNNs. As shown in Table 1, eight datasets are selected from UCI machine learning repository [58] to evaluate the performance of SOS. And six metaheuristic algorithms, including BBO [43], CS [37], GA [23], GSA [27, 28], PSO [28], and MVO [33], are presented for a reliable comparison.

### 5.1. Datasets Design

The Blood dataset contains 748 instances, which were selected randomly from the donor database of Blood Transfusion Service Center in Hsinchu City in Taiwan. As a binary classification problem, the output class variable of the dataset represents whether the person donated blood in a time period (1 stands for donating blood; 0 stands for not donating blood). And the input variables are Recency, months since last donation; Frequency, total number of donation; Monetary: total blood donated in c.c.; and Time, months since first donation [59].

The Balance Scale dataset is generated to model psychological experiments reported by Siegler [60]. This dataset contains 625 examples and each example is classified as having the balance scale tip to the right and tip to the left or being balanced. The attributes are the left weight, the left distance, the right weight, and the right distance. The correct way to find the class is the greater of (left distance *∗* left weight) and (right distance *∗* right weight). If they are equal, it is balanced.

Haberman's Survival dataset contains cases from a study that was conducted between 1958 and 1970 at the University of Chicago's Billings Hospital on the survival of patients who had undergone surgery for breast cancer. The dataset contains 306 cases which record two survival status patients with age of patient at time of operation, patient's year of operation, and number of positive axillary nodes detected.

The Liver Disorders dataset was donated by BUPA Medical Research Ltd to record the liver disorder status in terms of a binary label. The dataset includes values of 6 features measured for 345 male individuals. The first 5 features are all blood tests which are thought to be sensitive to liver disorders that might arise from excessive alcohol consumption. These features are Mean Corpuscular Volume (MCV), alkaline phosphatase (ALKPHOS), alanine aminotransferase (SGPT), aspartate aminotransferase (SGOT), and gamma-glutamyl transpeptidase (GAMMAGT). The sixth feature is the number of alcoholic beverage drinks per day (DRINKS).

The Seeds dataset consists of 210 patterns belonging to three different varieties of wheat: Kama, Rosa, and Canadian. From each species there are 70 observations for area *A*, perimeter *P*, compactness *C*  (*C* = 4*∗pi∗A*/*P*^2^), length of kernel, width of kernel, asymmetry coefficient, and length of kernel groove.

The Wine dataset contains 178 instances recording the results of a chemical analysis of wines grown in the same region in Italy but derived from three different cultivars. The analysis determined the quantities of 13 constituents found in each of the three types of wines.

The Iris dataset contains 3 species of 50 instances each, where each species refers to a type of Iris plant (setosa, versicolor, and virginica). One species is linearly separable from the other 2 and the latter are not linearly separable from each other. Each of the 3 species is classified by three attributes: sepal length, sepal width, petal length, and petal width in cm. This dataset was used by Fisher [61] in his initiation of the linear-discriminate-function technique.

The Statlog (Heart) dataset is a heart disease database containing 270 instances that consist of 13 attributes: age, sex, chest pain type (4 values), resting blood pressure, serum cholesterol in mg/dL, fasting blood sugar > 120 mg/dL, resting electrocardiographic results (values 0, 1, and 2), maximum heart rate achieved, exercise induced angina, oldpeak = ST depression induced by exercise relative to rest, the slope of the peak exercise ST segment, number of major vessels (0–3) colored by fluoroscopy, and thal: 3 = normal; 6 = fixed defect; 7 = reversible defect.

### 5.2. Experimental Setup

In this section, the experiments were done using a desktop computer with a 3.30 GHz Intel(R) Core(TM) i5 processor, 4 GB of memory. The entire algorithm was programmed in MATLAB R2012a. The mentioned datasets are partitioned into 66% for training and 34% for testing [33]. All experiments are executed for 20 different runs and each run includes 500 iterations. The population size is considered as 30 and other control parameters of the corresponding algorithms are given below:

In CS, the possibility of eggs being detected and thrown out of the nest is *pa* = 0.25.
In GA, crossover rate *P*_*C*_ = 0.5; mutate rate *P*_*c*_ = 0.05.
In PSO, the parameters are set to *C*_1_ = *C*_2_ = 2; weight factor *w* decreased linearly from 0.9 to 0.5.
In BBO, mutation probability *p*_*m*_ = 0.1, the value for both max immigration (*I*) and max emigration (*E*) is 1, and the habitat modification probability *p*_*h*_ = 0.8.
In MVO, exploitation accuracy is set to *p* = 6, the min traveling distance rate is set to 0.2, and the max traveling distance rate is set to 1.
In GSA, *α* is set to 20, the gravitational constant (*G*_0_) is set to 1, and initial values of acceleration and mass are set to 0 for each particle.

All input features are mapped onto the interval of [−1, 1] for a small scale. Here, we apply min-max normalization to perform a linear transformation on the original data as given in (12), where *v*′ is the normalized value of *v* in the range [min⁡, max⁡].

(12) $$\begin{array}{c} v^{\prime }=2*\frac{v-\min}{\max-\min}-1. \end{array}$$

### 5.3. Results and Discussion

To evaluate the performance of the proposed method SOS with other six algorithms, BBO, CS, GA, GSA, PSO, and MVO, experiments are conducted using the given datasets. In this work, all datasets have been partitioned into two sets: training set and testing set. The training set is used to train the network in order to achieve the optimal weights and bias. The testing set is applied on unseen data to test the generalization performance of metaheuristic algorithms on FNNs.

Table 2 shows the best values of mean squared error (MSE), the worst values of MSE, the mean of MSE, and the standard deviation for all training datasets. Inspecting the table of results, it can be seen that SOS performs best in datasets Seeds and Iris. For datasets Liver Disorders, Haberman's Survival, and Blood, the best values, worst values, and mean values are all in the same order of magnitudes. While the values of MSE are smaller in SOS than the other algorithms, which means SOS is the best choice as the training method on the three aforementioned datasets. Moreover, it is ranked second for the dataset Wine and shows very competitive results compared to BBO. In datasets Balance Scale and Statlog (Heart), the best values in results indicate that SOS provides very close performances compared to BBO and MVO. Also the three algorithms show improvements compared to the others.

Convergence curves for all metaheuristic algorithms are shown in Figures 4, 6, 8, 10, 12, 14, 16, and 18. The convergence curves show the average of 20 independent runs over the course of 500 iterations. The figures show that SOS has the fastest convergence speed for training all the given datasets. Figures 5, 7, 9, 11, 13, 15, 17, and 19 show the boxplots relative to 20 runs of SOS, BBO, GA, MVO, PSO, GSA, and CS. The boxplots, which are used to analyze the variability in getting MSE values, indicate that SOS has greater value and less height than those of SOS, GA, CS, PSO, and GSA and achieves the similar results to MVO and BBO.

Through 20 independent runs on the training datasets, the optimal weights and biases are achieved and then used to test the classification accuracy on the testing datasets. As depicted in Table 3, the rank is in terms of the best values in each dataset and SOS provides the best performances on testing datasets: Blood, Seeds, and Iris. For dataset Wine, the classification accuracy of SOS is 98.3607% which indicates that only one example in testing dataset cannot be classified correctly. It is noticeable that, though MVO has the highest classification accuracy in datasets Balance Scale, Haberman's Survival, Liver Disorders, and Statlog (Heart), SOS also performs well in classification. However, the accuracy shown in GA is the lowest among the tested algorithms.

This comprehensive comparative study shows that the SOS algorithm is superior among the compared trainers in this paper. It is a challenge for training FNN due to the large number of local solutions in solving this problem. On account of being simpler and more robust than competing algorithms, SOS performs well in most of the datasets, which shows how flexible this algorithm is for solving problems with diverse search space. Further, in order to determine whether the results achieved by the algorithms are statistically different from each other, a nonparametric statistical significance proof known as Wilcoxon's rank sum test for equal medians [62, 63] was conducted between the results obtained by the algorithms, SOS versus CS, SOS versus PSO, SOS versus GA, SOS versus MVO, SOS versus GSA, and SOS versus BBO. In order to draw a statistically meaningful conclusion, tests are performed on the optimal fitness for training datasets and* P values* are computed as shown in Table 4. Rank sum tests the null hypothesis that the two datasets are samples from continuous distributions with equal medians, against the alternative that they are not. Almost all values reported in Table 4 are less than 0.05 (5% significant level) which is strong evidence against the null hypothesis. Therefore, such evidence indicates that SOS results are statistically significant and that it has not occurred by coincidence (i.e., due to common noise contained in the process).

### 5.4. Analysis of the Results

Statistically speaking, the SOS algorithm provides superior local avoidance and the high classification accuracy in training FNNs. According to the mathematical formulation of the SOS algorithm, the first two interaction phases are devoted to exploration of the search space. This promotes exploration of the search space that leads to finding the optimal weights and biases. For the exploitation phase, the third interaction phase of SOS algorithm is helpful for resolving local optima stagnation. The results of this work show that although metaheuristic optimizations have high exploration, the problem of training an FNN needs high local optima avoidance during the whole optimization process. The results prove that the SOS is very effective in training FNNs.

It is worth discussing the poor performance of GA in this subsection. The rate of crossover and mutation are two specific tuning parameters in GA, dependent on the empirical value for particular problems. This is the reason why GA failed to provide good results for all the datasets. In the contrast, SOS uses only the two parameters of maximum evaluation number and population size, so it avoids the risk of compromised performance due to improper parameter tuning and enhances performance stability. Easy to fall into local optimal and low efficiency in the latter of search period are the other two reasons for the poor performance of GA. Another finding in the results is the good performances of BBO and MVO which are benefit from the mechanism for significant abrupt movements in the search space.

The reason for the high classification rate provided by SOS is that this algorithm is equipped with adaptive three phases to smoothly balance exploration and exploitation. The first two phases are devoted to exploration and the rest to exploitation. And the three phases are simple to operate with only simple mathematical operations to code. In addition, SOS uses greedy selection at the end of each phase to select whether to retain the old or modified solution. Consequently, there are always guiding search agents to the most promising regions of the search space.

## 6. Conclusions

In this paper, the recently proposed SOS algorithm was employed for the first time as a FNN trainer. The high level of exploration and exploitation of this algorithm were the motivation for this study. The problem of training a FNN was first formulated for the SOS algorithm. This algorithm was then employed to optimize the weights and biases of FNNs so as to get high classification accuracy. The obtained results of eight datasets with different characteristic show that the proposed approach is efficient to train FNNs compared to other training methods that have been used in the literatures: CS, PSO, GA, MVO, GSA, and BBO. The results of MSE over 20 runs show that the proposed approach performs best in terms of convergence rate and is robust since the variances are relatively small. Furthermore, by comparing the classification accuracy of the testing datasets, using the optimal weights and biases, SOS has advantage over the other algorithms employed. In addition, the significance of the results is statistically confirmed by using Wilcoxon's rank sum test, which demonstrates that the results have not occurred by coincidence. It can be concluded that SOS is suitable for being used as a training method for FNNs.

For future work, the SOS algorithm will be extended to find the optimal number of layers, hidden nodes, and other structural parameters of FNNs. More elaborate tests on higher dimensional problems and large number of datasets will be done. Other types of neural networks such as radial basis function (RBF) neural network are worth further research.

## Figures and Tables

**Figure 4:**
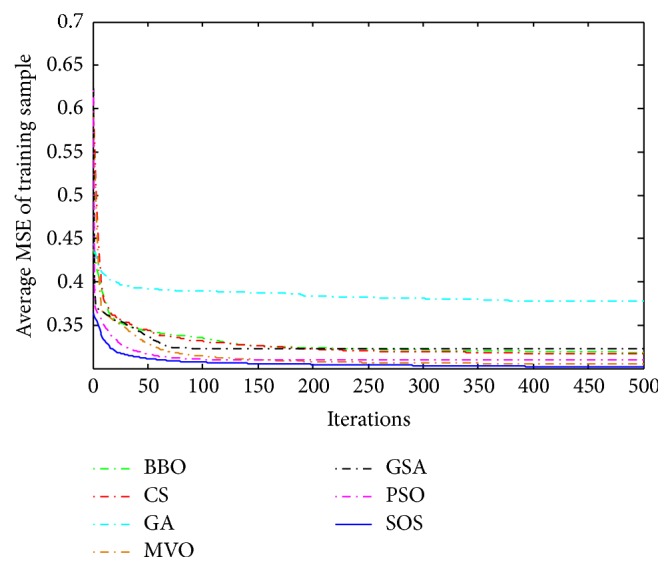
The convergence curves of algorithms (Blood).

**Figure 5:**
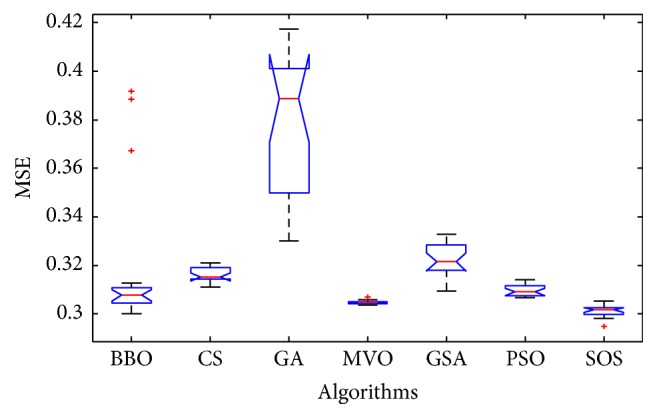
The ANOVA test of algorithms for training Blood.

**Figure 6:**
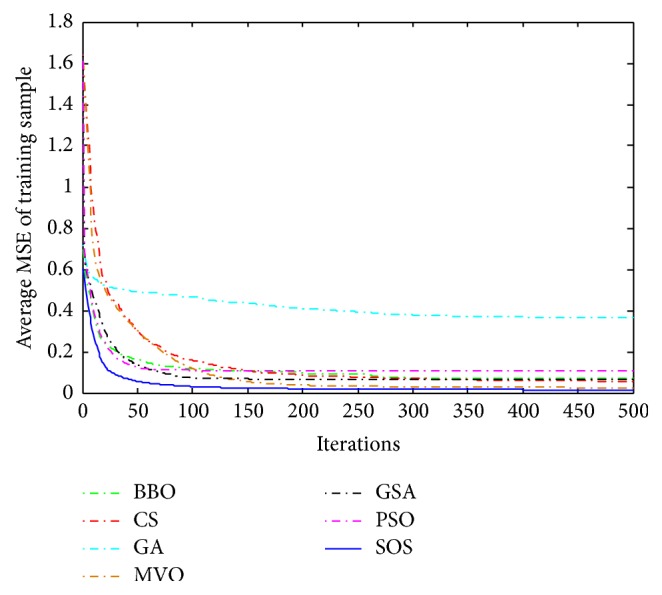
The convergence curves of algorithms (Iris).

**Figure 7:**
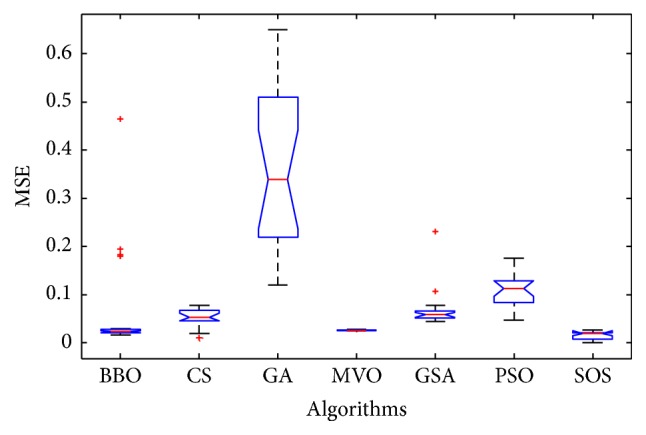
The ANOVA test of algorithms for training Iris.

**Figure 8:**
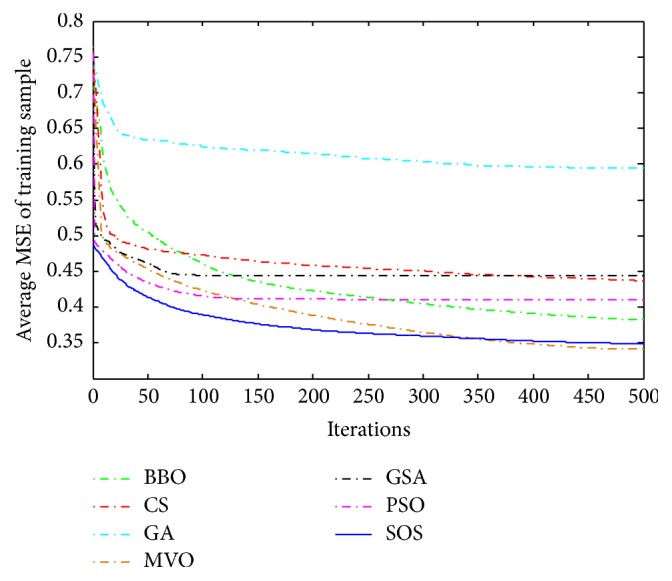
The convergence curves of algorithms (Liver Disorders).

**Figure 9:**
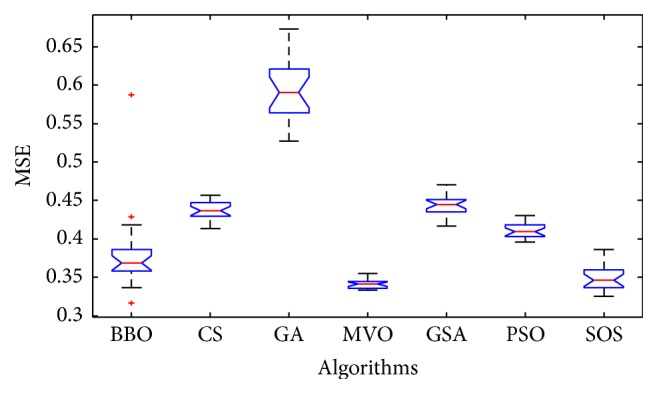
The ANOVA test of algorithms for training Liver Disorders.

**Figure 10:**
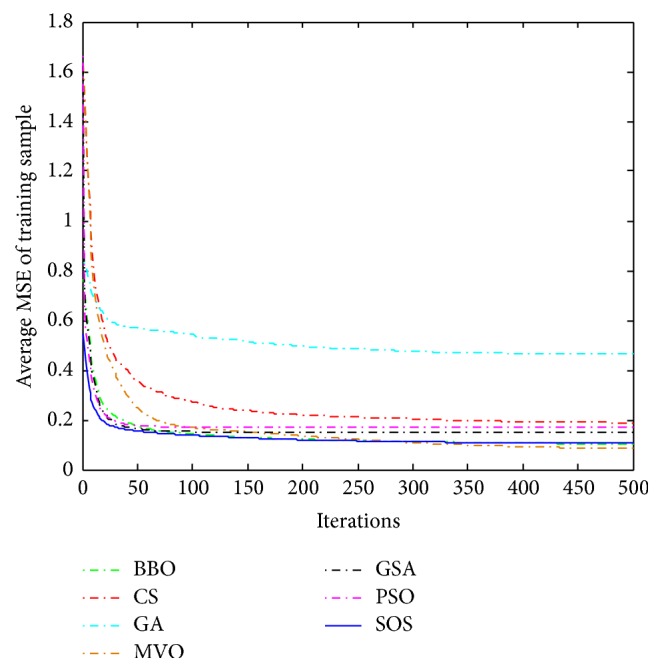
The convergence curves of algorithms (Balance Scale).

**Figure 11:**
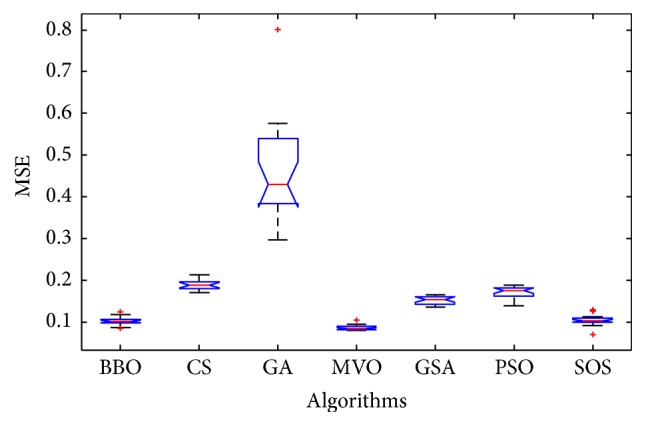
The ANOVA test of algorithms for training Balance Scale.

**Figure 12:**
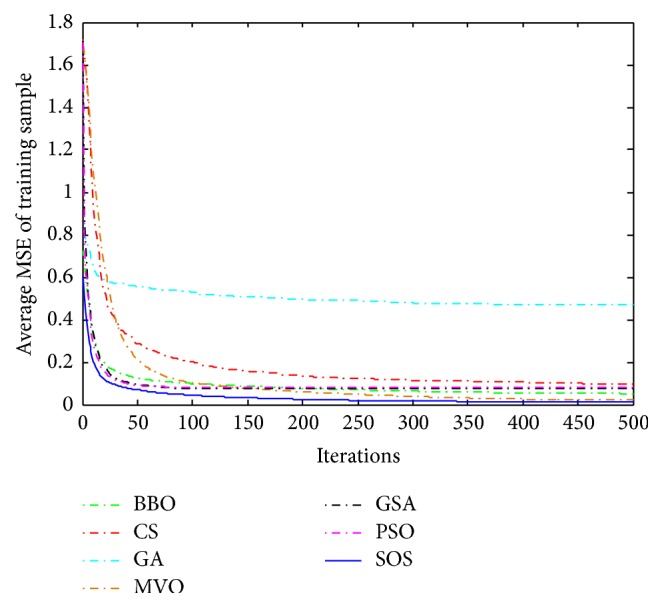
The convergence curves of algorithms (Seeds).

**Figure 13:**
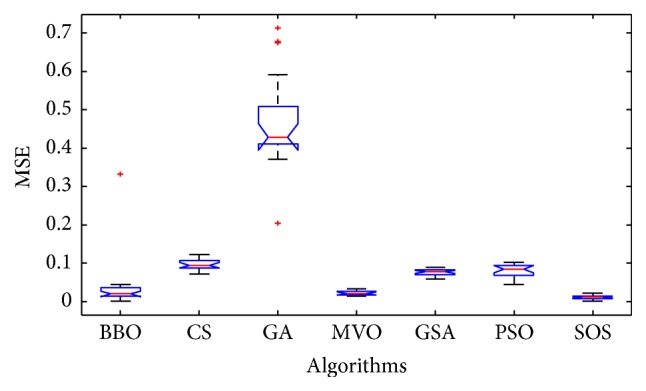
The ANOVA test of algorithms for training Seeds.

**Figure 14:**
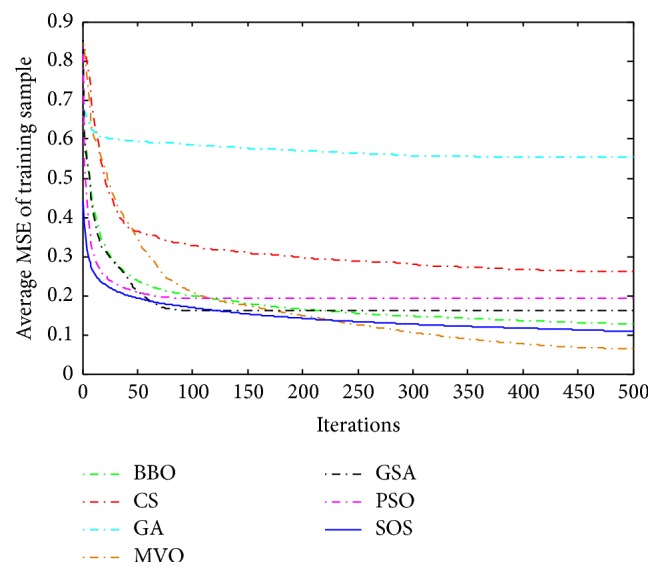
The convergence curves of algorithms (Statlog (Heart)).

**Figure 15:**
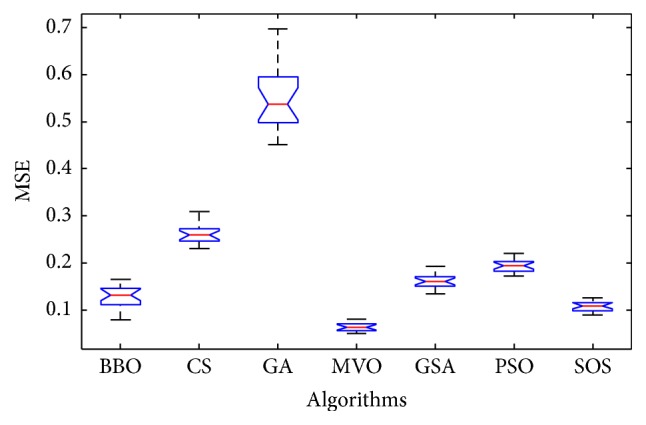
The ANOVA test of algorithms for training Statlog (Heart).

**Figure 16:**
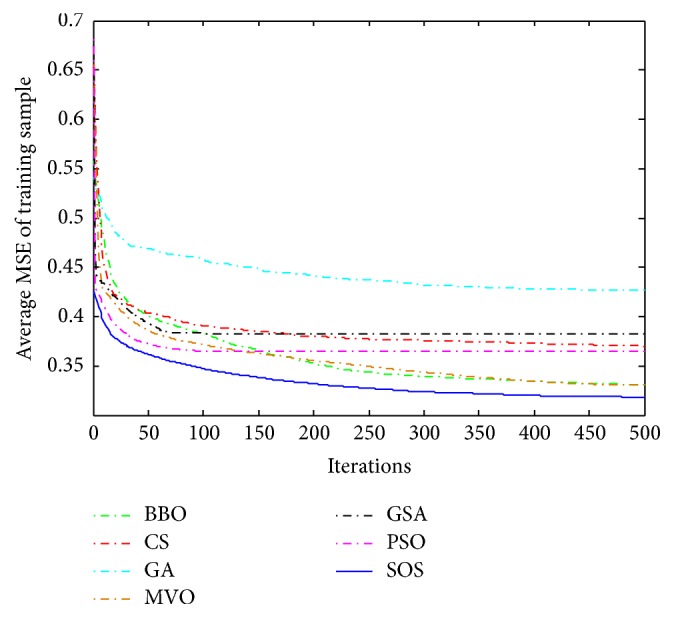
The convergence curves of algorithms (Haberman's Survival).

**Figure 17:**
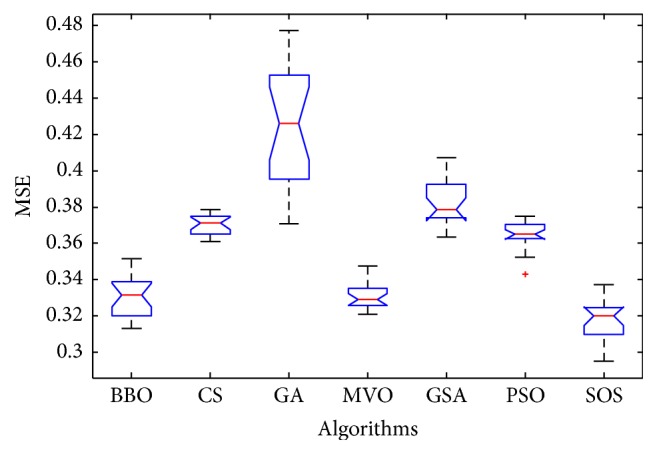
The ANOVA test of algorithms for training Haberman's Survival.

**Figure 18:**
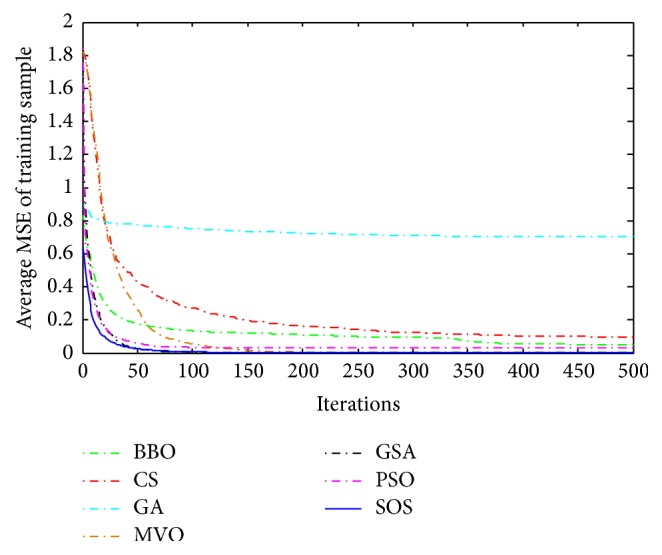
The convergence curves of algorithms (Wine).

**Figure 19:**
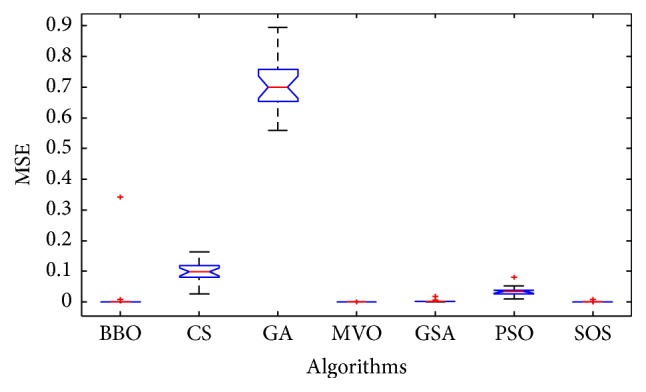
The ANOVA test of algorithms for training Wine.

**Table 1:** Description of datasets.

Dataset	Attribute	Class	Training sample	Testing sample	Input	Hidden	Output
Blood	4	2	493	255	4	9	2
Balance Scale	4	3	412	213	4	9	3
Haberman's Survival	3	2	202	104	3	7	2
Liver Disorders	6	2	227	118	6	13	2
Seeds	7	3	139	71	7	15	3
Wine	13	3	117	61	13	27	3
Iris	4	3	99	51	4	9	3
Statlog (Heart)	13	2	178	92	13	27	2

**Table 2:** MSE results.

Dataset	Algorithm						
	SOS	MVO	GSA	PSO	BBO	CS	GA
Blood							
Best	2.95*E* − 01	3.04*E* − 01	3.10*E* − 01	3.07*E* − 01	3.00*E* − 01	3.11*E* − 01	3.30*E* − 01
Worst	3.05*E* − 01	3.07*E* − 01	3.33*E* − 01	3.14*E* − 01	3.92*E* − 01	3.21*E* − 01	4.17*E* − 01
Mean	3.01*E* − 01	3.05*E* − 01	3.23*E* − 01	3.10*E* − 01	3.18*E* − 01	3.17*E* − 01	3.78*E* − 01
Std.	2.44*E* − 03	7.09*E* − 04	6.60*E* − 03	2.47*E* − 03	2.84*E* − 02	2.97*E* − 03	2.85*E* − 02

Balance Scale							
Best	7.00*E* − 02	8.03*E* − 02	1.37*E* − 01	1.40*E* − 01	8.52*E* − 02	1.70*E* − 01	2.97*E* − 01
Worst	1.29*E* − 01	1.04*E* − 01	1.66*E* − 01	1.88*E* − 01	1.24*E* − 01	2.14*E* − 01	8.01*E* − 01
Mean	1.05*E* − 01	8.66*E* − 02	1.52*E* − 01	1.72*E* − 01	1.02*E* − 01	1.89*E* − 01	4.65*E* − 01
Std.	1.33*E* − 02	6.06*E* − 03	9.46*E* − 03	1.32*E* − 02	9.45*E* − 03	1.18*E* − 02	1.16*E* − 01

Haberman's Survival							
Best	2.95*E* − 01	3.21*E* − 01	3.64*E* − 01	3.43*E* − 01	3.13*E* − 01	3.61*E* − 01	3.71*E* − 01
Worst	3.37*E* − 01	3.48*E* − 01	4.07*E* − 01	3.75*E* − 01	3.51*E* − 01	3.79*E* − 01	4.77*E* − 01
Mean	3.18*E* − 01	3.31*E* − 01	3.82*E* − 01	3.65*E* − 01	3.31*E* − 01	3.70*E* − 01	4.27*E* − 01
Std.	1.04*E* − 02	6.63*E* − 03	1.14*E* − 02	7.84*E* − 03	1.07*E* − 02	5.83*E* − 03	3.45*E* − 02

Liver Disorders							
Best	3.26*E* − 01	3.33*E* − 01	4.17*E* − 01	3.96*E* − 01	3.16*E* − 01	4.14*E* − 01	5.27*E* − 01
Worst	3.86*E* − 01	3.55*E* − 01	4.71*E* − 01	4.31*E* − 01	5.87*E* − 01	4.57*E* − 01	6.73*E* − 01
Mean	3.49*E* − 01	3.41*E* − 01	4.44*E* − 01	4.11*E* − 01	3.82*E* − 01	4.37*E* − 01	5.95*E* − 01
Std.	1.65*E* − 02	6.17*E* − 03	1.32*E* − 02	1.06*E* − 02	5.47*E* − 02	1.12*E* − 02	4.30*E* − 02

Seeds							
Best	9.78*E* − 04	1.44*E* − 02	5.97*E* − 02	4.49*E* − 02	2.07*E* − 03	7.19*E* − 02	2.05*E* − 01
Worst	2.26*E* − 02	3.30*E* − 02	8.87*E* − 02	1.02*E* − 01	3.32*E* − 01	1.22*E* − 01	7.14*E* − 01
Mean	1.11*E* − 02	2.21*E* − 02	7.65*E* − 02	8.17*E* − 02	5.23*E* − 02	9.80*E* − 02	4.71*E* − 01
Std.	5.36*E* − 03	6.31*E* − 03	8.82*E* − 03	1.53*E* − 02	9.63*E* − 02	1.34*E* − 02	1.20*E* − 01

Wine							
Best	6.35*E* − 11	1.34*E* − 06	5.87*E* − 04	9.74*E* − 03	5.62*E* − 12	2.56*E* − 02	5.60*E* − 01
Worst	8.55*E* − 03	2.91*E* − 05	1.90*E* − 02	8.03*E* − 02	3.42*E* − 01	1.62*E* − 01	8.95*E* − 01
Mean	4.40*E* − 04	5.41*E* − 06	3.21*E* − 03	3.39*E* − 02	5.17*E* − 02	9.59*E* − 02	7.03*E* − 01
Std.	1.91*E* − 03	6.23*E* − 06	3.96*E* − 03	1.55*E* − 02	1.25*E* − 01	3.48*E* − 02	8.59*E* − 02

Iris							
Best	5.10*E* − 08	2.45*E* − 02	4.41*E* − 02	4.71*E* − 02	1.70*E* − 02	1.01*E* − 02	1.19*E* − 01
Worst	2.67*E* − 02	2.75*E* − 02	2.31*E* − 01	1.76*E* − 01	4.65*E* − 01	7.84*E* − 02	6.51*E* − 01
Mean	1.42*E* − 02	2.58*E* − 02	6.83*E* − 02	1.09*E* − 01	6.91*E* − 02	5.32*E* − 02	3.66*E* − 01
Std.	8.80*E* − 03	8.40*E* − 04	4.07*E* − 02	3.55*E* − 02	1.11*E* − 01	1.93*E* − 02	1.70*E* − 01

Statlog (Heart)							
Best	8.98*E* − 02	5.03*E* − 02	1.35*E* − 01	1.72*E* − 01	8.03*E* − 02	2.31*E* − 01	4.52*E* − 01
Worst	1.26*E* − 01	8.13*E* − 02	1.92*E* − 01	2.20*E* − 01	1.65*E* − 01	3.09*E* − 01	6.97*E* − 01
Mean	1.09*E* − 01	6.48*E* − 02	1.62*E* − 01	1.93*E* − 01	1.27*E* − 01	2.61*E* − 01	5.53*E* − 01
Std.	1.07*E* − 02	9.11*E* − 03	1.47*E* − 02	1.32*E* − 02	2.37*E* − 02	1.86*E* − 02	7.20*E* − 02

**Table 3:** Accuracy results.

Dataset	Algorithm						
	SOS	MVO	GSA	PSO	BBO	CS	GA
Blood							
Best	82.7451	81.1765	78.0392	80.3922	81.1765	78.8235	76.8627
Worst	77.6471	80.0000	33.3333	77.6471	72.5490	74.5098	67.0588
Mean	79.8039	80.7451	74.1765	79.2157	77.5294	76.9608	72.7843
Rank	1	2	6	4	2	5	7

Balance Scale							
Best	92.0188	92.9577	91.0798	89.2019	91.5493	90.6103	80.2817
Worst	86.8545	89.2019	85.9155	83.5681	88.2629	82.1596	38.0282
Mean	90.0235	91.4319	87.7465	86.7136	90.1643	86.3146	59.7653
Rank	2	1	4	6	3	5	7

Haberman's Survival							
Best	81.7308	82.6923	81.7308	82.6923	82.6923	81.7308	78.8462
Worst	71.1538	75.9615	74.0385	76.9231	69.2308	74.0385	65.3846
Mean	76.0577	79.5673	79.1827	80.4808	77.0192	78.2692	74.5673
Rank	4	1	4	1	1	6	7

Liver Disorders							
Best	75.4237	76.2712	64.4068	72.0339	72.8814	67.7966	55.0847
Worst	66.1017	72.0339	6.7797	59.3220	45.7627	47.4576	27.1186
Mean	71.0593	74.1949	48.3051	66.4831	65.1271	56.1441	43.0508
Rank	2	1	6	4	3	5	7

Seeds							
Best	95.7746	95.7746	94.3662	92.9577	94.3662	91.5493	67.6056
Worst	87.3239	90.1408	85.9155	78.8732	61.9718	77.4648	28.1690
Mean	91.3380	93.4507	90.3521	87.4648	87.3239	82.2535	51.1268
Rank	1	1	3	5	3	6	7

Wine							
Best	98.3607	98.3607	100.0000	100.0000	100.0000	91.8033	49.1803
Worst	91.8033	96.7213	91.8033	88.5246	59.0164	78.6885	18.0328
Mean	95.4918	97.9508	96.1475	96.0656	90.4918	83.3607	32.6230
Rank	4	4	1	1	1	6	7

Iris							
Best	98.0392	98.0392	98.0392	98.0392	98.0392	98.0392	98.0392
Worst	64.7059	98.0392	33.3333	52.9412	29.4118	52.9412	7.8431
Mean	92.0588	98.0392	93.7255	91.4706	82.9412	85.0000	56.0784
Rank	1	1	1	1	1	1	1

Statlog (Heart)							
Best	85.8696	88.0435	86.9565	86.9565	80.4348	83.6957	70.6522
Worst	77.1739	77.1739	33.6957	77.1739	66.3043	68.4783	39.1304
Mean	82.2283	82.6087	79.1848	82.8804	75.5435	77.4457	50.5435
Rank	4	1	2	2	6	5	7

**Table 4:** *P values* produced by Wilcoxon's rank sum test for equal medians.

Dataset	SOS versus					
	CS	PSO	GA	MVO	GSA	BBO
Blood	6.80*E* − 08	6.80*E* − 08	6.80*E* − 08	3.07*E* − 06	6.80*E* − 08	4.68*E* − 05
Balance Scale	6.80*E* − 08	6.80*E* − 08	6.80*E* − 08	9.75*E* − 06	6.80*E* − 08	2.29*E* − 01
Haberman's Survival	6.80*E* − 08	6.80*E* − 08	6.80*E* − 08	1.79*E* − 04	6.80*E* − 08	2.56*E* − 03
Liver Disorders	6.80*E* − 08	6.80*E* − 08	6.80*E* − 08	1.26*E* − 01	6.80*E* − 08	1.35*E* − 03
Seeds	6.80*E* − 08	6.80*E* − 08	6.80*E* − 08	3.99*E* − 06	6.80*E* − 08	1.63*E* − 03
Wine	6.78*E* − 08	6.80*E* − 08	6.80*E* − 08	1.48*E* − 03	1.05*E* − 06	5.98*E* − 01
Iris	2.96*E* − 06	6.47*E* − 08	6.47*E* − 08	7.62*E* − 07	6.47*E* − 08	5.73*E* − 05
Statlog (Heart)	6.80*E* − 08	6.80*E* − 08	6.80*E* − 08	6.80*E* − 08	6.80*E* − 08	6.04*E* − 03
